# Multi-Organ Dysfunction in Cerebral Palsy

**DOI:** 10.3389/fped.2021.668544

**Published:** 2021-08-09

**Authors:** John Allen, Zunera Zareen, Samantha Doyle, Laura Whitla, Zainab Afzal, Maria Stack, Orla Franklin, Andrew Green, Adam James, Timothy Ronan Leahy, Shoana Quinn, Basil Elnazir, John Russell, Sri Paran, Patrick Kiely, Edna Frances Roche, Ciara McDonnell, Louise Baker, Owen Hensey, Louise Gibson, Stephanie Kelly, Denise McDonald, Eleanor J. Molloy

**Affiliations:** ^1^Discipline of Pediatrics, School of Medicine, Trinity College Dublin, The University of Dublin, Dublin, Ireland; ^2^Trinity Research in Childhood Centre, Trinity College Dublin, Dublin, Ireland; ^3^Children's Health Ireland (CHI) at Tallaght, Dublin, Ireland; ^4^St. Michael's House, Dublin, Ireland; ^5^Department of Clinical Genetics, Birmingham Women's Hospital, Birmingham, United Kingdom; ^6^Royal College of Surgeons in Ireland, Dublin, Ireland; ^7^Children's Health Ireland at Crumlin, Dublin, Ireland; ^8^Children's Health Ireland at Temple St. Dublin, Dublin, Ireland; ^9^Central Remedial Clinic, Dublin, Ireland; ^10^Department of Paediatrics, Cork University Hospital, Cork, Ireland; ^11^Department of Neonatology, The Coombe Women and Infants University Hospital, Dublin, Ireland

**Keywords:** cerebral palsy—complications, multi-organ dysfunction, cerebral palsy, multi-system involvement, review (article), neurological impairment, neurodevelopmental disorders, neurodisability

## Abstract

Cerebral Palsy (CP) describes a heterogenous group of non-progressive disorders of posture or movement, causing activity limitation, due to a lesion in the developing brain. CP is an umbrella term for a heterogenous condition and is, therefore, descriptive rather than a diagnosis. Each case requires detailed consideration of etiology. Our understanding of the underlying cause of CP has developed significantly, with areas such as inflammation, epigenetics and genetic susceptibility to subsequent insults providing new insights. Alongside this, there has been increasing recognition of the multi-organ dysfunction (MOD) associated with CP, in particular in children with higher levels of motor impairment. Therefore, CP should not be seen as an unchanging disorder caused by a solitary insult but rather, as a condition which evolves over time. Assessment of multi-organ function may help to prevent complications in later childhood or adulthood. It may also contribute to an improved understanding of the etiology and thus may have an implication in prevention, interventional methods and therapies. MOD in CP has not yet been quantified and a scoring system may prove useful in allowing advanced clinical planning and follow-up of children with CP. Additionally, several biomarkers hold promise in assisting with long-term monitoring. Clinicians should be aware of the multi-system complications that are associated with CP and which may present significant diagnostic challenges given that many children with CP communicate non-verbally. A step-wise, logical, multi-system approach is required to ensure that the best care is provided to these children. This review summarizes multi-organ dysfunction in children with CP whilst highlighting emerging research and gaps in our knowledge. We identify some potential organ-specific biomarkers which may prove useful in developing guidelines for follow-up and management of these children throughout their lifespan.

## Introduction

Cerebral palsy (CP) describes a heterogenous group of non-progressive disorders of posture or movement, causing activity limitation, due to a lesion in the developing brain (1). The worldwide prevalence of CP is ~2 per 1,000 live births (2), although the most recent data from the Surveillance of Cerebral Palsy in Europe Network reports the prevalence as 1.64 per 1,000 live births (3).

There are many recognized risk factors for cerebral palsy, including preterm birth, multiple gestation, congenital malformation, genetic and metabolic abnormalities, intra-uterine exposure to infection or inflammation, birth asphyxia, perinatal stroke and thrombophilia (4). CP has an overall rate of 13% in survivors of term neonatal encephalopathy (NE), and 24% of term children with CP have a history of moderate to severe NE (5). Neonatal asphyxia induces global hypoxic-ischaemia resulting in multi organ injury (6). Early multi-organ dysfunction (MOD) in neonates with encephalopathy may persist in later childhood. Cardiac, renal, hepatic, hematological and neurological dysfunction are well-described in neonates with NE, but follow-up in childhood is not routine practice.

Life-expectancy is reduced in children with CP, based on retrospective data collected between 1983 and 2010 in California (7, 8). Respiratory and cardiac dysfunction are well-documented causes of increased morbidity and mortality in children with CP and dysfunction in other organs such as the renal, gastrointestinal and hematological systems are gaining recognition. Assessment of multi-organ function may be useful to ensure complete resolution of any abnormalities and avoid complications in later childhood or adulthood. It may also contribute to an improved understanding of the etiology and thus may have an implication in prevention, interventional methods and therapies.

While CP is heterogenous condition with a wide range of etiologies and subtypes it is acknowledged that functional level is a strong predictor of morbidity and mortality. Greater degrees of motor impairment are associated with a larger proportion of accompanying impairments (9, 10). Mortality also increases with increasing severity of impairment and those with the highest disability scores have a 50% mortality by 15 years of age (11). Throughout this article, although some issues are present across all functional levels, the co-morbidities described are most-likely to be relevant to those with a Gross Motor Function Classification system (GMFCS) of IV-V, unless otherwise stated. Our focus is on children with CP, but as more children survive into adulthood we believe it is important to consider that the care provided in childhood may have a significant impact on future health. Also, where little research in children with CP exists, or, where the phenotype changes between childhood and adulthood, we have evaluated and included adult studies because we believe this can provide valuable insights.

We aimed to summarize multi-organ dysfunction in children with CP whilst highlighting emerging research and gaps in our knowledge. We identify some potential organ-specific biomarkers which may prove useful for research purposes or for developing guidelines for follow-up and management of these children throughout their lifespan (Table 1).

**Table 1:** Summary of multi-organ involvement, management and potential future directions for research in Cerebral Palsy (CP).

	**Possible considerations for care**	**Treatment and potential future directions**
Neurological	• MRI brain for all (12) • Cognitive impairment • Epilepsy • Behavioral issues, including inattention and hyperactivity • Spasticit	• Consider genetic and metabolic investigations if brain malformation, unclear etiology, or findings atypical (12) • Seizure management does not significantly differ from children without CP (13) • Medical options for spasticity management include diazepam, baclofen (oral or intrathecal) and botulinum toxin (14)
Hearing and vision	• Hearing impairment • Cortical Visual Impairment (CVI), refractive errors and accommodative dysfunction are common in CP (15, 16)	• Consider thorough audiological assessment if concerned • Screening questionnaires are available to help with recognition of CVI (15) • Early and periodic screening of vision recommended
Respiratory	• Significant cause of morbidity and mortality • Largest cause of premature death in children and young people with CP (17) • Dysphagia • Aspiration • LRTI more common and severe (18) • Bronchiectasis (19) SNI and GOR most significant risk factors	• Benefit of treating GOR for respiratory reasons unclear. Large prospective trials required • Mucolytics and physical therapies may help with tenacious secretions (20) • Prophylactic antibiotics – randomized controlled trials required
Sleep	• Sleep disorders • Association with seizures (21) • DIMS most common subtype (22) • Sleep disordered breathing more common in CP (21, 23)	• Screen for sleep disorders using validated tools • First-line therapy – sleep hygiene (24, 25) • Trial of melatonin where no identifiable cause of sleep disturbance found (13) • Adenotonsillectomy first-line treatment for OSA (26) • Consider non-invasive ventilation but high failure rate (27)
Cardiac	• Adults with CP have 3-fold increase in CVS disorders (28) • Altered inflammation and reduced activity are risk factors for endothelial dysfunction and later atherosclerosis (29–31).	• Long-term follow up studies required to demonstrate predictive value of CIMT and HRV on adult morbidity and mortality • Potential for use of serum biomarkers, such as Troponin, and advanced echocardiography to evaluate cardiac dysfunction in CP
Renal and urinary tract	• Lower urinary tract dysfunction (32) • Urinary incontinence most common • Bladder dysfunction may be linked with upper urinary tract deterioration • UTI more common in CP (33) • Higher risk of CKD (34)	• Blood pressure and urinary protein currently useful for monitoring renal function • Potential for novel biomarkers such as Cystatin-C, NGAL and IL-6 in the future but further research required in this population • Minimize nephrotoxic drugs • Low Creatinine may not be a reliable method of monitoring for renal dysfunction in CP
Gastrointestinal	• Dysphagia • Drooling • Diagnosis of GOR may be challenging due to communication difficulties • Nutritional optimisation associated with better functional status (35) • Constipation • Increased risk of acute and chronic abdominal pain which may be difficult to diagnose particularly if non-verbal (36, 37)	• Multi-disciplinary approach to management of drooling • Anticholinergic agents and intraglandular botulinum toxin are mainstay of medical treatment of drooling (38) • Use PPI as first line treatment of GORD with H2RAs as alternative. Consider surgical alternatives if medical treatment fails (39) • Annual monitoring of micronutrients (35) • Thorough assessment for any nociceptive sources of abdominal pain. If no source evident consider trial of gabapentin (40) • Further research required on gut failure in CP
Hematological	• Thrombophilias have been associated with neonatal stroke and hemiplegic CP (41) • Increased blood loss seen during spinal surgery (42) • Iron deficiency has a negative effect on functional ability and muscle strength (43)	• Consider prophylactic tranexamic acid and early use of blood products for surgical interventions (44) • Monitor for iron deficiency and anemia and treat accordingly
Inflammation and Infection	• Children post-neonatal encephalopathy have altered inflammatory responses which persist until school age (31). • Children with CP are at increased risk of all infections, including respiratory infections, post-operative infective complications and invasive pneumococcal disease (45–48)	• Further research is required to show whether children with CP have persistent inflammatory and immune dysfunction • Follow national immunization schedule, recommend influenza vaccination and consider extended-coverage polyvalent pneumococcal vaccine
Metabolic	• Metabolic disorders may mimic CP	• Consider metabolic testing if progression, developmental regression, atypical history or neuroimaging, positive family history (49)
Genetics	• Single gene disorders may produce a CP phenotype (50) • Genetic polymorphisms may increase susceptibility to developing CP (51)	• Further research will likely expand the number of genetic disorders and polymorphisms known to cause or increase susceptibility to CP • The epigenome provides promise as a therapeutic target to improve neurodevelopmental outcome in CP
Endocrine	• Increased risk of growth anomalies secondary to GH deficiency and nutritional deficiency (52) • Most common childhood condition associated with osteoporosis (53) • Early adrenarche is seen in CP but often not indicative of true central precocious puberty (54)	• Formal measurement of body proportions at least twice per year • If concerned re osteoporosis, perform DXA at 6 years and then biennially (53) • Evidence for treatment with bisphosphonates, vitamin D and calcium in CP but further work required on benefit of weight-bearing (55, 56) • Regularly assess puberty with Tanner staging (57)
Orthopedic	• Scoliosis – associated with poorer gross motor function (58) • Hip dislocation - associated with poorer gross motor function (59) • Upper limb contractures	• Hip surveillance -dependent on GMFCS • Further research on effectiveness of interventions to prevent hip dislocation essential • Level of evidence for interventions relating to the upper limb is lower and requires further study

*MRI, Magnetic Resonance Imaging; LRTI, Lower Respiratory Tract Infection; SNI, Severe Neurological Impairment; GOR, Gastro-esophageal Reflux; QoL, Quality of Life; DIMS, Disorders of Initiation and Maintenance of Sleep; OSA, Obstructive Sleep Apnoea; CVS, Cardiovascular; CIMT, Carotid Intima Media Thickness; HRV, Heart Rate Variability; UTI, Urinary Tract Infection; CKD, Chronic Kidney Disease; CVI, Cortical Visual Impairment; GH, Growth Hormone; DXA, Dual-energy X-ray absorptiometry; PPI, Proton Pump Inhibitors; H2RA, Histamine 2 Receptor Antagonists; GORD, Gastro-esophageal Reflux Disease*.

## The Nervous System

Cerebral palsy, by definition, is attributable to a lesion in the developing brain (1). Periventricular white matter lesions are observed on magnetic resonance images (MRI) in 50% of children with CP, and cortical or subcortical gray matter lesions in 20% (60). However, in a study of children with CP born after 36 weeks' gestation, neuroimaging studies were normal in one third of children (61). In the same study, the most common abnormality on neuroimaging was focal infarction, observed in 22% of the children. Other abnormalities noted were brain malformation, including schizencephaly, hydrocephalus, polymicrogyria, lissencephaly, agenesis of the corpus callosum, septo-optic dysplasia, and cerebellar anomalies. Periventricular leukomalacia (PVL) which is associated with prematurity was seen in 12% of the children, hypoxic ischaemic brain injury in 5% and intracranial hemorrhage in a further 5% (61). Gray and white matter lesion burden has been shown to correlate with motor and cognitive function (60). The American Academy of Neurology recommend neuroimaging in all children with CP. In those where history and imaging do not determine etiology or where findings are atypical, genetic or metabolic investigations should be considered. Where a brain malformation is found, it is recommended that genetic and metabolic conditions also be considered (12).

Cerebral Palsy is the most common cause of motor disability in children (4). Studies of the epidemiology of CP traditionally group children with CP into phenotypic categories based on the type of tone abnormality and the distribution of limb weakness (61). The severity of motor impairment is assessed by the Gross Motor Function Classification System (GMFCS) and this is the most widely used standard measure of motor function (62). Aside from motor impairment, neuropsychological & cognitive function is commonly impaired in children with CP. Intellectual disability is seen in approximately one third of patients with CP, GMFCS I-II and two thirds of patients with CP, GMFCS III-V (13) and has previously been shown to be the strongest predictor of mortality in CP (5). Children with CP who have a history of neonatal encephalopthy (NE) are significantly more likely to develop cognitive impairment and have a greater burden of disability, including epilepsy, than children with CP who did not have NE (5).

The overall prevalence of epilepsy in children with cerebral palsy was found to be 38%, although this varies significantly depending on CP subtypes, etiology and cognitive function (63). Prevalence increases with increasing severity of motor impairment. There is a higher prevalence of epilepsy in children with CP secondary to CNS malformations, CNS infections, and gray matter lesions than those with CP secondary to white matter changes or CP of unknown etiology. Furthermore, the prevalence of epilepsy in CP increased with decreased cognitive function (63). Age of onset of epilepsy can vary with type of CP, and early recognition and diagnosis is essential for prompt management. Management of seizure disorders are not significantly different in children with CP compared to those without, but care should be taken not to confuse seizures with dyskinesia (13). Behavioral issues are also more common in children with CP overall and especially with coexisting epilepsy (64). Hyperactivity and inattention are significantly higher in children with CP and epilepsy than in children with CP alone (64).

Hypertonia is a significant issue for many children with CP, leading to impaired motor function, pain and difficulties with daily care. Hypertonia may manifest primarily as spasticity, dystonia or less commonly rigidity, alone or in combination (65). Spasticity management involves a multi-disciplinary approach, including individual, goal-oriented physiotherapy and occupational therapy (14). Options for medical treatment include diazepam, baclofen (oral or intrathecal) and botulinum toxin type A (14). In certain cases, Selective Dorsal Rhizotomy may be considered (14). Dystonia management requires specific medications, including trihexyphenidyl and gabapentin, but current evidence is limited and the majority of care pathways rely on expert opinion (66, 67). Choice of treatment should be tailored to the individual and based on their treatment goals.

### Hearing and Vision

The incidence of hearing impairment in children with CP is reported to be between 7 to 37.5% (68) with the following distribution: 48% conductive, 4% sensorineural, 25% mixed and 23% unspecified (68). Conductive hearing loss has been associated with a high rate of chronic otitis media, eustachian tube dysfunction, abnormal anatomy and craniofacial anomalies in this cohort. Sensorineural hearing loss (SNHL) has been postulated to be associated with low birth weight, hyperbilirubinemia and neonatal hypoxemia in this population, with more severe hearing loss in those with quadriplegia, epilepsy or intellectual disability (68). Furthermore, congenital CMV infection is associated with cerebral palsy and is a leading cause of SNHL in children (69). Although there are inherent difficulties in testing this patient group for hearing loss using pure tone audiometry, it should not preclude thorough audiological assessment.

Children with cerebral palsy can be diagnosed with visual impairments that are ocular or cerebral in origin or combination of both (70). Cortical visual impairment (CVI), refractive errors and accommodative dysfunction are common in children with cerebral palsy (15). Periventricular Leucomalacia (PVL) on MRI is found to have a strong association with CVI (71). Dutton et al. provide a questionnaire inventory as an effective means to assist the clinician in recognition of CVI so that the appropriate strategies and management can be put in place to assist the child (15). Lesions involving the basal ganglia are more frequently associated with impaired visual function than those involving the visual occipital cortex (72). Furthermore, there is a correlation between the severity of basal ganglia lesions and the degree of visual impairment, with moderate and severe lesions of the basal ganglia always associated with visual impairment in one study (73). White matter lesions are not as reliable an indicator of neonates who will develop visual impairment, as only those with severe white matter changes had visual impairment and the presence or severity of visual impairment was not always associated with lesions involving the visual cortex. There was over 90% concordance between visual assessments performed in the 1st year of life and at school-going age (73), supporting the need for early and periodic screening and long-term monitoring.

## Respiratory Dysfunction

Disorders of the respiratory system are a significant cause of morbidity (74) and are the largest cause of premature death in children and young people with CP (17, 75, 76). Respiratory symptoms are common, particularly in those with a GMFCS level of V and are most prevalent at meal-times (77). Respiratory diagnoses account for a high proportion of hospital admissions in this population (78, 79) and many of these admissions may be predictable and modifiable (79). Blackmore et al. demonstrated that 12.1% of children and young people with CP were admitted to hospital due to respiratory illness over the course of 1 year (80). In the same period, 19.2% had been prescribed 2 or more courses of antibiotics for respiratory issues (80).

Reasons for the high rate of respiratory morbidity are complex, multifactorial and several factors, such as seizures, oromotor dysfunction, gastro-esophageal reflux and scoliosis, may coexist within the same individual (80, 81). Impaired airway clearance, due to respiratory muscle weakness, impaired coordination and poor positioning, in the context of poorer motor function, may predispose to infection and atelectasis. Bronchopulmonary dysplasia may be an issue in children with CP who were born prematurely (74, 82).

Swallowing is inextricably linked with respiratory dysfunction. It is a complex process involving the brainstem, multiple overlapping cortical areas, sensory input from the oropharynx and a high level of coordination between the central and enteric nervous systems (83). Chewing and swallowing problems are significantly associated with poorer gross motor function and intellectual ability (84) and impact on respiratory health. Dysphagia is common, occurring in 43% of all children with CP (83) and in children with severe generalized CP and intellectual disability can be as high as 99% (85). This can result in video fluoroscopically-proven aspiration in up to 70% of children with severe CP (86). Aspiration is a significant cause of respiratory symptoms and pneumonia in CP and can result from both direct aspiration from the oropharynx or indirect aspiration of gastric contents (87). Aspiration may result in pneumonia through introduction of pathogenic bacteria, alteration of the respiratory microbiome, promotion of airway inflammation or a complex interaction of all of these factors (88–92).

Lower respiratory tract infections (LRTI) in general are more common in children with CP and they have more severe disease requiring admission to the intensive care unit (18). Thorburn et al. reported that 89% of children with moderate to severe CP (*n* = 53, 90% with GMFCS ≥3) ventilated in the pediatric intensive care unit for 4 or more days carried abnormal bacteria flora, with *Pseudomonas* and *Klebsiella* being the most frequent. In children without CP (*n* = 257) the carriage rates of abnormal bacteria were 55% with 10% resistant bacteria. Almost half of these were resistant bacteria and the mortality rate was significantly higher amongst the group with CP compared those without CP (17 vs. 10%) (93). Bronchiectasis, which predisposes to recurrent LRTI, is reported in 67% of patients with CP presenting to a dysphagia clinic (94). Similarly, a retrospective study of 100 children with chronic pulmonary aspiration found that 66% had bronchiectasis, with the significant risk factors found to be severe neurological impairment (NI) (OR 9.45, 95% CI 2.05–43.6) and parent report of previous gastroesophageal reflux (OR3.36 95% CI 1.08–10.43). In the cohort of children with severe NI (*n* = 30), 93% had bronchiectasis (19).

Management of respiratory morbidity often requires a multifaceted approach. Those who care for children with CP must rule out aspiration when they present with respiratory symptoms. In particular, one must be cognisant of the fact that silent aspiration is more common in children with NI (95). In the respiratory context, some authors argue that gastroesophageal reflux (GOR) should be treated (94), presumably in an effort to reduce indirect aspiration of gastric contents. However, the evidence here is not conclusive. A randomized control trial of 38 children with asthma and GOR found no difference in respiratory symptoms or lung function between those treated with a proton-pump inhibitor (PPI) and placebo (96). Conversely, a prospective study comparing PPI vs. histamine H2-receptor antagonists (H2RA) identified 35 out of 74 children with esophageal and extra-esophageal (respiratory and laryngeal) manifestations of GOR (97). Eighty-three percent had complete resolution of symptoms when treated with a PPI, compared with 35% in the H2RA group. Conflicting evidence is also seen with regards to the surgical treatment of GOR for respiratory symptoms. A prospective cohort study, comparing outcomes of Nissen fundoplication in children with NI (*n* = 46) vs. without NI (*n* = 42) showed a reduction in the proportion of children with NI who had more than 4 respiratory infections per year from 33% pre-operatively to 8, 18, and 10% at 1, 2, and 3 year postoperative, respectively (98). However, respiratory symptoms were not reported to be improved post-operatively. Another retrospective review of 34 patients following fundoplication, 15 of whom had NI, evaluated outcomes at 7 years post-operative. Pulmonary symptoms were frequent in children with NI pre-operatively (13/15) and did not significantly improve after surgery, while half of the children with NI developed new symptoms or complications during the period of follow-up (99). A retrospective analysis of discharge data for 342 pediatric patients, found no difference in hospital admissions for aspiration pneumonia, other pneumonia or respiratory distress before or after anti-reflux surgery (100). Finally, a meta-analysis by Lauriti et al. showed that gastro-jejunal feeding was no better at preventing respiratory complications than anti-reflux surgery in children with NI (101). Large prospective, high quality trials would be useful to determine if and when treatment of GOR is useful in improving respiratory morbidity.

The British Thoracic Society (BTS) recommends the use of mucolytics, such as nebulised saline, to assist clearance of “tenacious secretions” in children with neuromuscular weakness (20). Physical therapies to help removal of respiratory secretions are likely to be helpful but may be a significant extra burden on families (94). Finally, there is no evidence for the use of prophylactic antibiotics in children with CP. Long-term azithromycin is recognized as beneficial in cystic fibrosis (102) likely secondary to its anti-inflammatory properties. The BTS recommend consideration of prophylactic antibiotics in non-cystic fibrosis bronchiectasis although the authors acknowledge that controlled trials are lacking (103). Prophylactic antibiotics are frequently prescribed for children with CP. Macrolide antibiotics have antibacterial, anti-inflammatory, pharmacokinetic and bioavailability characteristics that make them useful for prophylaxis, as well as being safe with few side effects (74, 94, 104). High quality randomized controlled trials are required to assess their effectiveness and indications for use in children with CP.

### Sleep Problems

The prevalence of sleep disorders among children with CP is up to 36%, although this varies within subgroups, according to CP phenotype (bilateral spastic, 41%; unilateral spastic, 24%; dyskinesia, 30.8%; ataxia/non-classified, 17.4%) (105, 106) and can have a significant impact on quality of life (107). Total sleep disorders are principally associated with seizures (21). Subtypes of sleep disorders in children with CP include disorders of initiating and maintaining sleep (DIMS), sleep breathing disorders, sleep hyperhidrosis, sleep-wake transition disorders, disorders of arousal, parasomnias and non-restorative sleep (105). DIMS are the most frequently occurring subtype of sleep disorder with symptoms including difficulty falling asleep and frequent night awakening (21, 22). This may be secondary to pain, involuntary movements, associated behavioral and adaptive difficulties, underlying brainstem dysfunction or severe visual impairment (21, 108–112).

Behavioral considerations are a significant cause of sleep disturbance in CP. In a study of children with moderate to severe motor disability (*n* = 505, 216 had CP) which used parental questionnaires to evaluate sleep problems, 25% of children with CP had difficulties relaxing at night (113). A systematic review and meta-analysis by Horwoord et al. (105) calculated the rate of problems with “sleeping routines” as 51.3%, using the data from a retrospective observational study of 154 children with CP. Interestingly, this concern decreased with worsening motor function from 100% in children classified as GMFCS I to 34% in children classified as GMFCS V and with increasing age.

The Sleep Disturbance Scale for Children (SDSC) (114, 115), Pediatric Sleep Questionnaire (PSQ) (116) and Childhood Sleep Habit Questionnaire (CSHQ) (117, 118) are validated tools used to document sleep problems in children and are frequently used in children with CP. Sleep affects the quality of life of these children (107, 119) and their families (120, 121) thus, sleep disorders are ideally evaluated in children with CP using these questionnaires and subsequently monitored and managed appropriately. Sleep hygiene is first-line therapy for sleep disturbance in all children including those with neurodevelopmental disabilities (24, 25, 122). This includes measures such as a consistent bedtime routine, a dark and quiet bedroom, limiting technology use before bed and independence in falling asleep (25, 123). Other therapy options for managing sleep disorders in children with neurodisability include behavioral strategies such as play-based therapy (124) and medications such as melatonin.

Recent research has provided interesting insights into some of the possible pathophysiological mechanisms that underlie sleep disturbance in CP. Circadian Locomotor Output Cycles Kaput (*CLOCK*) genes are rhythmically expressed in the pineal gland which secretes melatonin and is crucial for regulation of the sleep-wake cycle (125). Expression of *CLOCK* is altered following hypoxic-ischaemic brain injury in animal models (126) and alterations in circadian rhythm have been reported in children following even mild perinatal hypoxic-ischaemia (127). Day and night variability of melatonin has been found to be lower in children with CP (128). Melatonin is widely prescribed for sleep disturbances and has shown improvements in sleep latency and night-waking in these children (121, 129, 130). The National Institute for Health and Care Excellence (NICE) have produced a guideline on assessment and management of cerebral palsy in under 25-year-olds, within which guidance on managing sleep disturbances is provided (13). Sleep hygiene is recommended in all cases and melatonin where an identifiable cause of sleep disturbance is not found (13).

Sleep-disordered breathing is more common in children with CP than in their typically-developing peers (21, 23, 131), occurring in up to 18.1% of children with CP compared with 7.4% of controls (109). Children with more severe CP as measured by the GMFCS, as well as children with comorbid epilepsy have a higher risk of obstructive sleep apnoea (OSA) (132). OSA may be contributed to by hypotonia, glossoptosis, laryngomalacia, midface and mandibular abnormalities, adenotonsillar hypertrophy, gastro-esophageal reflux, medications and brainstem dysfunction (23, 27). Children with CP may be at increased risk of central sleep apnoea (CSA) as neurological disorders are the commonest risk factor for this (133). Sleep-disordered breathing in CP may also be caused by muscular weakness. Children with CP have reduced diaphragmatic mobility, respiratory muscle strength and chest expansion (134). Sleep disorders in general have a significant impact on quality of life in CP (107, 135) and therefore, treatment should be strongly considered for sleep disordered breathing. Non-invasive ventilation is a potential treatment. However, this can prove more challenging in children with CP and in a retrospective review, 55% of patients with CP failed to establish on this treatment compared with an overall failure rate of 8.7% in the same tertiary service (27). The American Academy of Pediatrics has recognized adenotonsillectomy as first line treatment for sleep apnoea in children (136). A randomized trial (*n* = 464) showed benefits in reducing symptoms of OSA as well as improvements in behavioral, quality of life and polysomnographic measures (26). Surgery may be rendered more difficult in children with neurological impairment due to co-morbidities. A retrospective review of 375 children with OSA and syndromic or neurological comorbidities, 105 of whom had cerebral palsy, found that the average apnoea-hypopnoea index reduced from 12.4 to 5.7 (*p* = 0.002) following tonsillectomy, leading the authors to conclude that adenotonsillectomy remained the mainstay of treatment for OSA in these children.

Sleep disorders can be difficult to diagnose in this population but can have a significant effect on the lives of children with CP and their families. It is important to be mindful of sleep issues and consideration should be given to regular screening for these disorders in children with CP.

## The Cardiovascular System

A cohort study which included 958 adults with CP reported that they had a 3-fold increase in disorders of the circulatory system (28). In a linkage study involving the Victorian Cerebral Palsy Register and the Australian National Death Index, out of 3,507 individuals with CP, 418 were known to have died between the years 1970 and 2010, with cardiac causes of death second only to respiratory (76).

Chronic inflammation is known to contribute to the formation of atherosclerosis, a common cardiovascular disorder (29) and children with CP are known to have altered inflammatory responses (30, 31) increasing the possibility that these children with CP may develop arterial pathology that leads to atherosclerosis in adult life. Children with CP are less physically active than their typically developing peers (137) and lower levels of physical activity are more likely to be associated with endothelial dysfunction (138–140). Early stage atherosclerosis can be demonstrated by measuring the carotid intima media thickness (CIMT) of the carotid arteries using advanced ultrasound (141). Increased CIMT is predictive of atherosclerosis and future cardiovascular events (142). Carotid ultrasound assessment of children with CP (*n* = 100), demonstrated increased CIMT when compared to controls (*n* = 35), suggesting that children with CP are at increased risk of atherosclerosis and coronary artery disease (141). Follow up studies of these children in later childhood and adulthood are required for prompt diagnosis and management.

Heart rate variability (HRV) reflects autonomic control of the sinus node. Significant reductions in HRV have been found between children with CP and controls. Within children with CP, HRV significantly decreases with poorer motor function as measured by GMFCS (143–145). This indicates a less efficient and less adaptive cardiac autonomic system in children with CP (144) and reiterates the need for cardiac surveillance in those with CP, particularly those most severely affected.

Measurement of fetal HRV has also been raised as a possible tool for predicting the development of cerebral palsy. A retrospective review of 95 children with CP, born at term, compared data from fetal monitoring and compared them with controls. Late decelerations and reduced beat-to-beat variability were associated with a sharp increase in risk of developing CP (146). Reduction in HRV can improve the prediction of neurodevelopmental outcome in preterm infants (*n* = 79) when combined with poor repertoire abnormal general movements (PR GMs) using Prechtl's assessment method (147, 148).

Evidence for myocardial dysfunction in children with CP is lacking. However, the possibility of myocardial involvement is raised by the fact that a proportion of neonates with NE have concurrent cardiac dysfunction (149). Martin-Ancel et al. showed that 29% of neonates with perinatal asphyxia had cardiac dysfunction consistent with myocardial ischaemia (150). The rate of cardiovascular system involvement in infants with post-asphyxial hypoxic ischaemic encephalopathy is reported to be between 50 and 78% (151–153). The variation may be due to the difference in criteria used for involvement of cardiac dysfunction in the above studies. Children with epilepsy who are seizure free have been shown to have impaired systolic and diastolic cardiac function using 2-dimensional speckle tracking echocardiography (154). In this case-control study (*n* = 120) there were no differences between the subgroups to explain this subclinical dysfunction and suggest that there may be unknown factors involved. Krishnamoorthy et al. prospectively assessed myocardial dysfunction in patients with moderate to severe traumatic brain injury using speckle tracking echocardiography and compared them with age and sex-matched controls (*n* = 30) (155). They have shown that abnormalities of myocardial strain are seen in this population and that the abnormality persists for at least 1 week. The mechanism underlying this is uncertain but may relate to neuroendocrine dysfunction and dysregulated inflammation (156).

Although it is known that there is acute myocardial dysfunction at the time of the initial insult in some children who develop CP, the long term myocardial function of these patients is not known. Traditional markers of cardiac function such as ejection fraction/shortening fraction are too crude to recognize potential subtle signs of myocardial dysfunction and are unable to detect regional wall dysfunction. With the advancement of echocardiography, newer forms of assessing myocardial function are readily available such as deformation imaging using speckle tracking, tissue doppler, tricuspid annular plane systolic excursion, mitral annular plane systolic excursion and fractional area change. These more sophisticated echocardiography techniques may be able to detect early cardiac dysfunction helping to guide management of these complex patients.

There is a paucity of research in follow-up of cardiovascular dysfunction throughout childhood in cerebral palsy. Echocardiographic assessment of myocardial strain combined with serum biomarkers may allow more accurate evaluation of cardiac dysfunction in later childhood. Troponin T and N-terminal pro b-type natriuretic peptide (NT-pro-BNP) have been shown to be useful markers of cardiac dysfunction in other populations, such as term neonates with perinatal asphyxia, children with chronic kidney disease and children with leukemia (157–159). A prospective study of infants with perinatal asphyxia (*n* = 54) showed that Troponin T is elevated in those with abnormal MRI findings and that Troponin T correlates with developmental outcomes at 2 years of age (160). Further research is required to establish whether initial abnormal Troponin T and BNP results correlate with subtle myocardial dysfunction parameters in later childhood.

## Renal and Urinary Tract Dysfunction

Renal and urinary tract dysfunction is multifactorial in the population of children with CP. Lower urinary tract dysfunction and renal dysfunction may be linked. Lower urinary tract dysfunction is more common in those with CP, affecting, on average 55.5% of this population, urinary incontinence being the most common symptom (32). Lower urinary tract symptoms (LUTS) are known to have a significant impact on quality of life in children and their families (161, 162). Urinary incontinence is more common in children and adults with CP (163–165). Children with CP achieve continence later than their typically developing peers (165, 166) and experience more daytime incontinence, nocturnal enuresis, or a combination of both (165). Abnormal urodynamic studies have been reported in 85% of children with CP referred for urological assessment (*n* = 27) in the following categories: spastic diplegia (*n* = 10), spastic quadriplegia (*n* = 7), spastic hemiplegia (*n* = 5), dystonic quadriplegia (*n* = 3), dystonic diplegia (*n* = 1), spastic monoplegia (*n* = 1) and intellectual disability (*n* = 15) (167). The commonest presenting symptom was incontinence (74%) followed by frequency (56%) and urgency (37%) (167). Disorders of urinary storage, most frequently reduced bladder capacity, detrusor overactivity and increased post-void residual volumes, are more common than disorders of voiding (e.g., urinary hesitancy and frequency) (167–169). Greater degrees of motor impairment as evidenced by the GMFCS are associated with a greater frequency of lower urinary tract symptoms and urodynamic abnormalities (170). GMFCS and intellectual ability have both been associated with attainment of continence (165, 171) although agreement on the influence of intellectual ability on continence in CP is not universal (172). Reid et al. advocated for early investigation of urological symptoms with urodynamic studies in children with CP as a way to rationalize treatment, noting that in many cases incontinence can be improved or cured (167).

Bladder dysfunction such as interrupted voiding, hesitancy and urinary retention, but not urinary incontinence, have been linked with upper urinary tract deterioration in children with CP (173). Further urological evaluation may thus be warranted in these children. Notably, obstructive voiding complaints are more common in adults with CP than children and may represent progression over time (174). Half of adults with CP had small, high pressure bladders, putting them at risk for upper urinary tract deterioration (*n* = 49, mean age = 31 years, 55% male, 98% GMFCS III-V), thus emphasizing the need for ongoing monitoring of people with CP and urinary tract symptoms (174). That said, this paper describes the quarter of patients with CP who underwent urodynamic studies according to the centre's criteria for investigation, which includes recurrent UTI, progressive urinary incontinence, hydronephrosis, urinary retention and progressive LUTS. Therefore, there may be an element of selection bias which skews the results.

Urinary tract infections are more common in children with CP (33, 166) occurring in ~20% (175). Children with CP have an increased incidence of constipation and fecal incontinence (165, 176, 177), both risk factors for the development of UTI (178, 179). Recurrent febrile urinary tract infections have been linked with upper urinary tract deterioration in children with CP and should prompt further urological evaluation. Congenital urinary tract abnormalities have not been found to be more common in children with CP (180).

A significant number of children with CP post NE may develop renal dysfunction later in life. Up to 72% of infants who have a 5-min Apgar score of ≤ 6 show signs of renal compromise (181, 182). Renal involvement in the neonatal period is also associated with neurological severity and outcome (183–186). An episode of acute kidney injury (AKI) in the neonatal period is correlated with an increased risk of developing chronic kidney disease (CKD) in later life (181, 187, 188). Therefore, any children who have had AKI as a result of perinatal asphyxia, regardless of severity, require regular renal function and blood pressure monitoring.

In a large retrospective cohort study, adults with neurodevelopmental disabilities (*n* = 33,561) had a greater incidence rate of CKD than adults without neurodevelopmental disabilities (*n* = 6.5 million). This increased risk was maintained even when adjusted for demographics, diabetes, hypertension and cardiovascular disease. Adults with neurodevelopmental disabilities, throughout all subgroups, were also more likely to have advanced CKD. The incidence rate ratio of advanced CKD for those with CP compared to adults without NDDs was 1.83 (95% CI = 1.61–2.07) (34). This highlights that children with CP have a greater risk of developing CKD and renal function monitoring including minimizing nephrotoxic drugs should be considered.

One of the most widely used methods of monitoring renal function is to measure serum creatinine but it does not rise until there is significant renal dysfunction. In addition, children with non-ambulatory CP have been shown to have lower serum creatinine concentrations at baseline (189). This likely represents a reduction in muscle mass and bone mineral density which is evident in children with CP (190–192). This may present some difficulty in monitoring for CKD as these children may have falsely reassuring creatinine levels, compounded by the fact that early CKD is often silent. In the outpatient setting, markers such as blood pressure, along with urinary protein can be useful for monitoring renal function.

Novel biomarkers such as Neutrophil gelatinase-associated lipocalin (NGAL), cystatin C, Interleukin-6 (IL-6) and Fibroblast Growth Factor (FGF-23) can improve the early diagnosis of AKI and CKD (193–197), predict clinical outcome (198, 199) and help quantitation of severity in CKD (200). One or more of these or other novel biomarkers may prove useful for monitoring of renal function in children and adults with CP but this has not, as yet, been assessed in this population.

## Gastrointestinal Dysfunction

There is a high prevalence of gastrointestinal disorders in children with cerebral palsy including dysphagia, gastroesophageal reflux disease (GORD) and chronic constipation (201). Dysphagia is related to inefficiencies in the oral preparation, oropharyngeal and esophageal stages of swallowing. It is a common problem in children with CP, occurring in 43% (83), and can be caused by oromotor dysfunction, oral sensory impairment, abnormal neurological development and esophageal dysmotility. GORD in these children can make dysphagia worse (85). Clinically significant oral motor dysfunction was demonstrated in a further study in 90% of children with CP (202). Complications of dysphagia in this population include recurrent lower respiratory tract infections and chronic aspiration, as discussed previously, as well as malnourishment, with an association between dysphagia and poor weight gain (85).

The issue of drooling is inextricably linked with dysphagia and GORD and is defined as “the unintentional loss of saliva and contents from the mouth” (203). It occurs in between 37.4 and 58% (204) of children with cerebral palsy and is subdivided into anterior and posterior types (205). Anterior drooling occurs when saliva leaks from the mouth and posterior drooling is where the saliva spills into the oropharynx and hypopharynx (205). Children with cerebral palsy do not have excessive salivation, but an oromotor muscle incoordination and sensory perception issues (206). Severe anterior drooling can lead to social isolation, damp clothing, irritated facial skin, interference with speech, damage to books, communication aids and computers (203, 207). Posterior drooling can lead to aspiration which may result in pneumonia.

Sialorrhoea can be exacerbated by GORD in children with CP through the oesophagogastric reflex. Treatment of children with CP, siallorhoea and gastro-esophageal reflux with antireflux medication has been shown to reduce drooling in a double-blind, placebo-controlled, crossover trial (*n* = 9) (208).

A multidisciplinary approach to the management of drooling is advisable (209, 210). The team can include neurologists, otolaryngologists, pediatricians, plastic surgeons, speech and language therapists, psychologists, physiotherapists, nurses and teachers. The treatment options vary from less invasive behavioral and non-medical to pharmacological interventions to more invasive surgical management usually reserved for children over 4 years of age.

The two main medical treatments are anti-cholinergic agents and intraglandular botulinum toxin injections to the submandibular and/or parotid glands. The anti-cholinergic drugs most commonly used are atropine, benztropine, glycopyrrolate bromide and scopolamine. Pharmacological interventions cannot selectively block stimulation of the salivary glands and as a result unwanted side effects can occur for example, constipation, blurred vision behavior disturbance, urinary retention and thickening of secretions (211).

Botulinum Toxin Injections into the submandibular glands and/or parotid glands under ultrasound guidance is often used if there is an inadequate response to the anticholinergic drugs. The main drawback is that they are only effective for about 6 months, need to be repeated and responsiveness can diminish over time. Parents should be advised of the potential for adverse effects such as xerostomia and dysphagia (205). There is insufficient evidence on the efficacy of many of these pharmacological treatments in siallorhoea (204), however, benztropine and Botulinum Toxin injections have been shown to reduce drooling in a recent meta-analysis (38).

Surgery is usually reserved for children that have failed all the less invasive treatments discussed. Submandibular or parotid duct ligation as well as submandibular gland excision are not without significant complications, although rare. For example, salivary stones, scarring, facial and hypoglossal nerve damage (212, 213) can occur. Submandibular gland duct relocation is useful for anterior drooling (214) but contraindicated for posterior drooling.

Symptoms suggestive of GOR are seen in 77% of children with CP (*n* = 58), of whom 90% were subsequently proven to have GORD by pH monitoring and endoscopic evidence of oesophagitis (201). The increased risk of GORD in children with CP is due to poor posture, scoliosis, increased intra-abdominal pressure from spasticity or medication side effects. These side effects include those of anticonvulsants which may increase nausea, vomiting and dysphagia, and, in turn, worsen the severity of GORD (215). Prospective analysis of 101 children with NI demonstrated that early onset of NI, mitochondrial disorders and EEG abnormalities were significant risk factors for GOR (216). In a cohort of children with CP and GORD (*n* = 18), significantly prolonged gastric emptying and abnormal esophageal motility was demonstrated on manometry (*p* < 0.01), suggesting that GI motility dysfunction contributes to GOR and oesophagitis in this group (201).

The diagnosis of GOR in some children with CP may be challenging because of difficulties with communication. GOR has been found in 91% of children with CP who have regurgitation, vomiting, recurrent abdominal pain, haematemesis and/or pulmonary aspiration (201). However, in many cases these symptoms may not be present. Considerable agitation and autoaggressive behavior can be a marker for pathological GOR and therefore, should be excluded when parents report these behaviors (217).

The North American and European Society for Pediatric Gastroenterology Hepatology and Nutrition (NASPGHAN, ESPGHAN) have published guidelines on the treatment of GORD, based on the available evidence (39). Lifestyle factors such as raising the head of the bed and avoiding exposure to smoke may be helpful in treating GOR. Thickening of feeds can help to reduce overt regurgitation and vomiting (39). The mainstays of pharmacological management are histamine receptor antagonists (H2RA) and proton pump inhibitors (PPI). It is recommended that PPIs are used as first line pharmacologic treatment of GORD, with use of H2RAs if PPIs are not available or contra-indicated (39). Baclofen is an alternative which may be considered prior to surgery if other medical treatments have failed (39). Surgical interventions, such as fundoplication, can be considered in children with GORD and neurological impairment if optimal medical therapy has been unsuccessful, with total oesophagogastric dissociation reserved as a rescue procedure for those in whom fundoplication has failed (39).

Children with more severe CP are at greater risk of poor nutritional status (218). It is estimated that up to 46% of children with CP can be classified as undernourished (219), however, it is only documented in 7.9% of children with CP admitted to hospital, suggesting significant under recognition (220). Dysphagia, GORD and constipation were significantly associated with malnourishment (220). ESPGHAN has published detailed recommendations for monitoring and managing nutritional status in children with NI, including biannual monitoring of anthropometric measurements and annual measurement of micronutrients. Nutritional optimisation may be beneficial in improving the functional status of children with CP (35). In a prospective study (*n* = 14) where children with CP and malnourishment completed a 6 month nutritional rehabilitation programme, 64% had an improvements in their “Gross Motor Function Measure” scores (221).

Constipation is also a substantial problem in children with neurodevelopmental disabilities (222) affecting almost ¾ of children with CP (201). This is mainly due to prolonged transit time, particularly in the more proximal segments of the colon (201). Treatment of constipation for children with NI is not significantly different than their typically developing peers apart from avoidance of polyethylene glycol and liquid paraffin in those with significant risk of pulmonary aspiration. Additionally, increased fluid and fiber intake can be considered in this population (223).

Children with NI are at significant risk of acute and chronic pain with the abdomen accounting for a significant proportion (11–32%) of these symptoms (36, 37). In a cross-sectional study involving children with CP aged 5–18 years (*n* = 288), 67.1% had acute pain and 31.4% had chronic pain with risk factors including dyskinesia, bilateral involvement and GMFCS IV-V (224) and, of those with acute pain, 42% also reported chronic pain (224). Abdominal pain may occur as a result of nociceptive pain sources such as appendicitis, pancreatitis or cholecystitis, all of which may be more difficult to diagnose in children with CP, particularly if they are non-verbal. Children with CP are also at increased risk of pain as a direct result of neurological dysfunction, including central neuropathic pain and visceral hyperalgesia (36, 225). This is particularly seen with lesions in the thalamus and spinothalamic tract. In these situations, normal or minimally noxious stimuli can result in significant pain (226). This means that routine activities, such as feeding, which inevitably produce gut distension, may provoke considerable pain. Non-pharmacological approaches to treatment such as reduction in feed volumes, venting of feeding tubes or alterations in feed timing may be useful. Management of pain, including abdominal pain, in these children should initially begin with a thorough assessment for any nociceptive sources of pain. If no obvious source can be identified a trial of empiric medical treatment with gabapentin has been suggested, with escalation to tricyclic antidepressants and methadone considered the following steps (40). In two retrospective studies (*n* = 22 and *n* = 42), gabapentinoids have been shown to reduce pain behaviors (227, 228). Prospective trials to confirm efficacy in this cohort would be useful. Above all, a systematic and thorough approach to assessing and managing pain in these children is required.

Gut and intestinal failure is increasingly being recognized in children with neurodisabling conditions (229). This is not surprising given the sizeable interface between the central and enteric nervous system. However, there is a significant paucity of research in this area which should be addressed in the future.

## The Hematological System

There is evidence to suggest that cerebral infarction secondary to pre or perinatal cerebral occlusion occurs in 13-37% children with hemiplegic CP and the infarction may occur due to a thrombophilia (12, 41, 230). Gunther et al. (41) studied 91 cases of neonatal arterial-ischaemic stroke and found that 68% had at least one pro-thrombotic tendency. However it is important to note that 24% of the 182 controls also had an abnormality. Mercuri et al. noted that the factor V Leiden mutation in particular may be associated with poorer outcomes in neonatal stroke (230). Coagulopathies and thrombophilias have been reported to be associated with neonatal stroke include antiphospholipid antibodies (231), anti-cardiolipin antibodies (232), increased lipoprotein (41), protein C resistance (41) and protein C and S deficiencies (12). The American Academy of Neurology and Child Neurological Society have recommended that diagnostic testing for a coagulation disorder should be considered in children with hemiplegic CP although the level of evidence was relatively low (12). The NICE guidelines for assessment of cerebral palsy (13) make no recommendation for routine testing for coagulation or thromboembolic disorders in children with CP.

The coagulation and inflammatory systems overlap and interact. Activated coagulation factors are proinflammatory and in turn inflammation can promote coagulation (233). Elevated inflammatory markers and coagulation factors coexist in neonates who subsequently develop CP (233). In children with NE, a risk factor for CP, it is known that coagulation parameters are strong predictors of outcome (234). Antenatally, it has been shown that children who subsequently develop CP are more likely to have had 2 or more placental lesions of thrombosis or inflammation (235) and that fetal thrombotic vasculopathy is significantly associated with NE (236). It is likely, therefore, that the interplay between coagulation and inflammation contribute to white matter damage and multi-organ dysfunction (237) and are thus important in the etiology of CP (233).

Beyond the neonatal period, children with CP have prothrombin times (PT) and activated partial thromboplastin times (APTT) which are within the normal ranges but which are significantly longer than controls (42). They also have significantly reduced calcium and magnesium levels, both important cofactors for coagulation (42). Brenn et al. examined bleeding in children with severe spastic quadriplegia during posterior spinal fusion surgery compared with controls (*n* = 34) and found that children with CP have significantly more bleeding and that bleeding occurs earlier in the course of surgery (42). Kannan et al. also reported significantly more blood loss during spinal surgery in children with neuromuscular scoliosis compared to those with idiopathic scoliosis (*n* = 25) and found reduced factor VII activity (238). Children with CP are also often taking medication which can effect coagulation, as reported in several case reports, such as valproate (239, 240), carbamazepine (241), and azithromycin (242). Therefore, consideration of coagulopathy and thrombophilia in children with CP is important, particularly prior to surgery, including the administration of prophylactic tranexamic acid and early use of blood products (44).

There is a high rate of nutritional deficiency amongst children with CP (243–245), which may correlate with anemia. However, evidence regarding anemia in children with CP is sparse. In institutionalized children and young people (*n* = 108, age 8–29 years) with cerebral palsy, iron-deficiency was reported in 38% and anemia was found in 33.3% of participants. Both iron-deficiency and anemia were significantly increased in those who had liquid diets compared to normal diets (95.6 vs. 22.3% and 87 vs. 18.8%, respectively) (246). In another case series (*n* = 30) iron-deficiency anemia was found in 4 (13.3%) children with neuromotor disabilities (247). In comparison, rates of iron-deficiency anemia in both low and middle-income children in the United States of America have been reported to range between 2 and 3% (248, 249). This is significant because iron-deficiency, even without anemia, is known to affect neurodevelopment including motor, cognitive and social-emotional function (43). In children with spastic CP, it has been reported that iron deficiency anemia has a negative effect on functional ability and muscle strength (250). Further research is needed to quantify the extent of iron deficiency and anemia in children with CP to ensure adequate treatment if necessary.

## The Immune System—Inflammation and Infection

NE accounts for around 24% of all cases of CP in term infants (4, 5). Infants with NE have been reported to have persistent inflammatory response over the 1st week of life, with higher neutrophil, monocyte and Toll-like receptor-4 expression, correlating with the degree of brain injury (251, 252). High concentration of Interleukin (IL)-1, 8, 9 and tumor necrosis factor (TNF)-α were demonstrated in neonatal blood from children with Cerebral Palsy in comparison with control children (253). One of the most important underlying pathophysiological mechanisms leading to CP includes intra-amniotic inflammation and infection eliciting an inflammatory response and damage to the developing brain. Fetal inflammatory response syndrome (FIRS) is a severe form of intra amniotic infection or inflammation. It stimulates the activation of innate immune system of the fetus, similar to that seen in adult inflammatory response syndrome. FIRS can lead to multi-organ dysfunction and causes neurological, renal and hematological abnormalities (254).

Perinatal inflammation is also associated with many neuropsychiatric and neuro-psychological disorders and it is suggested that inflammation has long term consequences on the brain during childhood (255). Children post-NE have been shown to have persistent inflammatory responses at school age (31). The injury processes can persist for months and years and a tertiary mechanism of damage, which includes inflammation and epigenetic changes, has been proposed (255). Dysregulated immune function is also found in school age children with CP who had brain injury in the neonatal period (30). School-age, preterm children with CP secondary to PVL had significantly higher levels of TNF-α in peripheral blood mononuclear cells (PBMC's) after lipopolysaccharide (LPS) stimulation compared to control school-age preterm children with normal neurodevelopment.

Infections are a significant contributor to childhood death, particularly in those with underlying conditions. CP was the most common underlying condition associated with death due to infection in the 1–4 year age category (45). Pneumonia in particular is an important cause of morbidity and mortality in children with CP, especially among those with severe spastic quadriplegia, epilepsy and intellectual disability (46). An observational data-linkage study of a developmental anomaly registry with a national death index revealed that, of those with available causes of death, 58.6% were attributable to respiratory causes, of whom 49% died of pneumonia at a mean age of 14.6 years (11). Non-respiratory infections accounted for a further 5% of deaths at a mean age of 16.6 years.

Children with CP also appear to be at increased risk of infective complications following surgery. In a retrospective analysis of 1,250 children with CP who underwent appendectomy had a significantly greater risk of developing sepsis/organ failure (adjusted OR = 2.47; *p* = 0.13), operation-related infections (adjusted OR = 2.65; *p* = 0.001), pneumonia (adjusted OR4.26; *p* < 0.001), and urinary tract infection (adjusted OR = 5.19; *p* < 0.001) (47). The mechanisms underlying this increased risk are unknown and likely multifactorial.

Vaccination is one of the most successful ways of preventing infections. Children with CP should follow the vaccination schedule in the country in which they live. However, additional vaccinations may be recommended to provide them with added protection given their associated co-morbidities. Children with CP have a 2-3 times higher risk of incomplete or delayed immunization (256) A retrospective review of data from children <7 years of age included in the Victorian CP register (*n* = 476) found that 19.2% were overdue at least one vaccine (256). It has also been reported that children with moderate to severe motor impairment are less likely to have received all of their vaccinations than those with less severe impairment (*n* = 98; *p* < 0.05) (257).

Children with neurological disorders have been acknowledged as a population which is at increased risk of serious complications of influenza (258). Despite this only 74% of pediatricians in the United States recognized CP as a high-risk condition (*n* = 412) (259). This survey-based study also found that 50% of children with a neurologic or neurodevelopmental disorder (*n* = 1143) were already vaccinated or their parents planned for vaccination against influenza (259) and programmes to improve the uptake rate are required.

A large population-based, case-control study in Denmark (*n* = 1,665) shows a higher risk of invasive pneumococcal disease amongst children with several chronic conditions. The adjusted risk ratio for children with a neurological disorder compared with children without such a disorder was 3.0 (95% confidence interval = 2.1–4.3) (48). There may be an argument for providing these children with extended-coverage polyvalent pneumococcal vaccines but this requires further research.

Recent research has shown that inflammatory responses in children with CP are altered. Further research may help to clarify the role of inflammation as a tertiary cause of neurological injury with the potential to develop therapeutic targets. There may also be a significant impact on immune function and further work is needed to optimize vaccination programs for children with CP and improve infection-associated morbidity and mortality.

## Metabolic Considerations in Assessing MOD

Inborn errors of metabolism (IEM) can present with CP like symptoms. A systematic review by Leach et al. identified almost 70 IEMs belonging to 13 different biochemical categories, that closely mimic cerebral palsy (CP). While these only account for 0.1–0.2% of CP cases, early diagnosis is essential to prevent organ damage (260). Metabolic investigations should be considered where the clinical history is consistent with a metabolic disorder. These features may include chronic progression, neurodevelopmental regression or non-central nervous system (CNS) organ involvement (49). Other signs include absent history of perinatal injury, and pattern of disease inheritance; so called “familial CP” which can be elicited by obtaining a thorough family history and/or presence of involuntary movements, ataxia, muscle atrophy, oculomotor abnormalities or sensory loss (260–262). In children with CP and normal magnetic resonance imaging (MRI), further testing for metabolic and/or genetic conditions has been recommended (263, 264). The presence of abnormal MRI findings which are unexpected or inconsistent with the suspected etiology should also warrant metabolic investigations (263). An example of this are globus pallidus abnormalities which can be indicative of the rare neurometabolic disorder, neurodegeneration with brain iron accumulation (NIBA) with pantothenate kinase-associated neurodegeneration (PKAN) the most frequent subtype identified (265).

Individualized investigations for IEMs can be planned according to the individual clinical picture i.e., history, examination and any neuroimaging. These investigations may include plasma, urine or cerebrospinal amino acids, a plasma acylcarnitine profile and urine organic acids, mucopolysaccharides, and oligosaccharides (261, 266). Targeted biomarker analysis or single gene analysis are useful where the phenotype and investigative results highly correlate with a specific metabolic condition. For example a clinical picture of significant dystonia, macrocephaly and MRI revealing widened Sylvian fissures and basal ganglia abnormalities may be confirmed as glutaric aciduria type 1 with urine organic acid analysis and genetic study of the glutaryl CoA dehydrogenase (GCDH) gene (261, 262). However due to the overlapping and, in certain instances, unspecific phenotypes of IEMs, next generation and whole exome sequencing has proven useful in non-specific findings where no distinct etiology is suspected (260, 261). Due to the sheer number of IEMs and the generalized diagnostic approach currently used, Metabolomics has emerged as an innovative method of improving diagnostic efficiency of IEMs as there are numerous possible diagnoses and it may be potentially used as a tool in precision medicine (267, 268). It should be used in conjunction with next generation and exome sequencing to help clarify the pathogenicity of genetic variants.

Mitochondrial disorders are neurometabolic diseases which can also present with CP like symptoms (260, 262). They are characterized by dysfunctional energy production and typically manifest as multi-organ dysfunction, often with neurological impairment and can be similar to CP (261). Mitochondria are postulated to play a significant role in hypoxic-ischaemic events which may lead to CP and mitochondrial targets are now being explored as a potential future therapeutic intervention as an alternative to therapeutic hypothermia in those with perinatal brain injury. These targets include protecting from mitochondrial permeabilization, directly targeting mitochondrial downstream apoptotic pathways and indirect protection and preservation of mitochondrial function (269).

It is important to be mindful of potential metabolic diagnoses when seeing children with CP as they can have significant implications, the most significant of which is to prevent deterioration and improve clinical outcomes.

## Genetic Considerations in Assessing MOD

An increasing number of studies implicate genetics in the etiology of cerebral palsy (CP). A genetic component to cerebral palsy may prompt the clinician to be particularly vigilant for specific multi-organ abnormalities. Several non-metabolic single gene disorders can present as CP. Many single gene mutations linked with CP have been identified using whole exome sequencing, including mutations in *KCNC3, KANK1, AP4MI, GAD1*, and *ADD3* with different mutations linked to the development of different subtypes of CP (50, 270–272). For example, mutations in KCNC3 have been found in some individuals diagnosed with ataxic CP (50) and heterozygous deletions of *KANK1* are associated with early motor delay and hypotonia, progressing to spastic quadriplegia and intellectual disability (273). Each of these single gene disorders are individually rare but together may account for a significant number of cases of CP. They are important to recognize as a genetic diagnosis can help with prognosis, monitoring and family counseling.

It is clear there is no single “CP gene” but there is increasing recognition of a genetic element to CP in a large number of cases. Despite improvements in obstetric, prenatal and perinatal care, there has been little change in the incidence of CP in term neonates, which may be due to an underlying genetic pathophysiology (270, 273). In most studies the prevalence of CP appears to be higher in the male population compared to the female population and it has been suggested that recessive X-linked chromosome variants may contribute to this difference (270). Higher rates of CP have also been reported in monozygotic twins and consanguineous families (271). Furthermore, the presence of coexisting congenital anomalies is significantly higher in the CP population compared to their healthy counterparts with rates of 11–32 and 1–2%, respectively (270, 271).

A significant number of candidate CP genes have also been identified alongside other genetic polymorphisms which have been proposed to contribute to the etiology of CP including copy number variants and single nucleotide polymorphisms (271). *De novo* and inherited genetic variants may account for up to 30% of CP cases and have been postulated to, in some cases, directly cause CP, while in others merely contribute to CP susceptibility (270, 274). For example, a large Australian case-control study of children with CP (*n* = 443) found that polymorphisms in Toll Like Receptor-4 (TLR-4) reduced the risk of developing CP, while polymorphisms in IL-6 and IL-8 may increase susceptibility to CP in the presence of viral exposure (51).

Finally, epigenetic modifications are gaining increasing recognition as contributing to tertiary brain damage following an initial insult (275). Epigenetics refers to any process by which gene activity is altered without changing the DNA sequence, including methylation and histone, acetylation or micro-RNA modifications (276, 277). It is thought that early life exposures can modify the epigenome and provide a link to neurodevelopmental outcome (278, 279). Epigenome-wide analysis of 15 monozygotic twins discordant for CP showed regional differences in DNA methylation associated with development of CP (280). Gene loci involved were associated with hypoxia, inflammation and cell adhesion. Further research with larger sample sizes would be useful to confirm these findings. Differences in methylation were also noted to be significantly different between adolescents with CP and controls (*n* = 32, *p* < 0.05) (281). In the future, the epigenome may have potential as a therapeutic target to improve neurodevelopmental outcomes in CP.

The vast genetic heterogeneity underlying CP emphasizes the complexity of the contribution of genetics to the etiology and development of CP and points toward a multifactorial etiology of CP with interaction between genetics and the environment (271, 282). While genetic causation should not change the clinical diagnosis of CP, inclusion as a subclassification by etiology may allow for more targeted therapy in these cases (273, 274).

## The Endocrine System

All children with disability are at risk for some form of endocrine dysfunction due to the disruption of endocrine feedback secondary to abnormalities in muscle, bone or brain mechanisms. Regular assessment from an endocrine perspective should be considered with the emphasis on growth, pubertal status and bone health. Knowing the underlying etiology of cerebral palsy can focus endocrine evaluation but is not essential.

All children with cerebral palsy should have formal measurement of body proportions at least twice per year to guide nutrition and to identify growth anomalies. Assessing length or height in proportion to weight is important to ascertain whether short stature is due to nutritional deficiency or a hormone imbalance. Nutritional deficiency is more likely to result in delayed puberty. Exogenous obesity can drive growth in childhood but following puberty can result in obesity in an immobile child causing long term implications for respiratory function and sleep apnoea. Children with midline defects will have hypothalamic pituitary axis dysfunction which can result in growth hormone deficiency impacting on tone, mood and glycaemic control as well as stature. Studies have indicated that circulating GH secretion profiles are lower in children with cerebral palsy compared to controls (52, 283). Hypoglycaemia can occur as a consequence of metabolic stress in the newborn period, growth or cortisol hormone deficiency. Deranged glucose metabolism may only become apparent at times of stress and illness and in children with spasticity and seizure disorders could be missed.

CP is the most common childhood condition associated with osteoporosis, and children with CP frequently sustain fractures with minor trauma (53). Any child or adolescent with immobilization, nutritional deficiency (especially impacting vitamin D or calcium metabolism) or delays in pubertal onset will be impacted by a reduction in bone mass accrual. Apparent reduction in bone density may also be artifactual if not adjusted for height. Growth hormone has been shown to be positively associated with surrogate markers of bone turnover in puberty (284) suggesting that this could be a factor in the attainment of bone density due to the lower circulating levels of growth hormone (283). In a systematic review of children with severe cerebral palsy, significant determinants of low BMD were limited ambulation, feeding difficulties, previous fracture, anticonvulsant use, and lower fat mass (285). The International Society of Clinical Densitometry has identified the importance of correct bone mineral density assessment in cerebral palsy and has issued official positions on both the definition of osteoporosis in this condition (286) and the appropriate site of BMD assessment using bone densitometry (287). If a child with CP is considered at risk of osteoporosis, bone densitometry (DXA) scans should be performed to assess baseline at 6 years of age, and repeated every 1–2 years depending on individual risk factors (53). In treating children with CP and osteoporosis there is probable evidence for bisphosphonates, possible evidence for vitamin D and calcium supplementation and insufficient evidence for weight bearing activities as effective interventions to improve BMD (55, 56). Bisphosphonates have been shown to improve pain on manipulation in children with CP and osteoporosis (288).

Puberty also plays a role in the development of bone health. Clinical assessment of puberty with Tanner staging is therefore recommended (57). Mean age of breast development in girls with CP is similar but with wider range of onset, while Menarche occurs later in Caucasian girls with CP (289). Early adrenarche is seen in cerebral palsy but more commonly in those with hydrocephalus or associated epilepsy. This is not indicative of central precocious puberty and can progress slowly over many years before true pubertal onset (54). Many children and adolescents with cerebral palsy can experience normal pubertal progression and menses (290).

## The Musculoskeletal System

Scoliosis and hip dislocation are common problems in children with CP. The overall incidence of scoliosis in CP of 20–25% (291), with a risk of up to 90% for spinal deformities in patients with GMFCS level V (58). Scoliosis is related to poor truncal tone and muscle weakness (292) and predictors for scoliosis include GMCFS IV and V, epilepsy and limited knee extension (293). The scoliosis pattern in children with GMFCS IV is usually a single long C- shaped curve that is most often kyphotic, but sometimes lordotic. In ambulatory patients with less motor involvement the pattern of deformity may be similar to that in idiopathic scoliosis with both thoracic and lumbar components (292, 294). Progression of scoliosis is usually gradual, but onset of puberty, deterioration in neurological function or prolonged time spent in a wheelchair can lead to a more rapid progression (292). Progressive scoliosis causes difficulties with daily care and mobilization, can lead to pain, cardiac and pulmonary complications, altered seizure thresholds and skin compromise (295).

Hip dislocation develops in 15–20% of cases (59). There is almost a direct link between higher levels of GMFCS and risk of hip subluxation. Risk of developing a hip migration index (MI) >30% is ~30, 50, and 80% at GMFCS III, IV, and V, respectively (296). Rate of progression of the MI increases from almost 2% at GMFCS III to 12% at GMFCS V (296). Hip dislocation develops because of contractures and spasticity of adductors, hamstrings and hip flexors (297). The combination of this spasticity and reduction in weight bearing lead to acetabular dysplasia (298). Hip surveillance is considered essential in reducing hip dislocation and need for surgery (299). Hippotherapy may lead to improved symmetry and stability although grade of evidence is low (299). Splints may reduce rate of dislocation but are ineffective at preventing hip dislocation (299). There is insufficient evidence as to whether therapy with botulinum toxin can prevent hip dislocation (300).

Both of the aforementioned orthopedic complications of CP may be linked. Hip dislocation or windswept deformity of the hip many cause pelvic obliquity and trigger scoliosis, while scoliosis itself can lead to pelvic obliquity and thus increase the risk of hip dislocation especially on the high side (59). Early identification of these issues of scoliosis and hip dislocation are important for improved surgical outcomes, although there are multiple prerequisites before consideration of surgery, such as Cobb angle >40–50 degrees which is progressive and interfering with sitting, age and medical comorbidities (292).

The upper limb may also develop significant complications in CP. Upper limb contractures developed in 34% of a population-based sample of children with CP (*n* = 771). These contractures began at preschool age and the best predictor was high scores on the Manual Ability Classification System (301). The level of evidence for interventions relating to the upper limb is much lower than that for the lower limbs. Further high quality studies in this area are required.

## Discussion

Multi-organ dysfunction in cerebral palsy to date has not been quantified. Better insight into multi organ dysfunction in children with CP may be gained by using novel biomarkers and new diagnostic tools. In this review, we focused on various long-term issues associated with each organ system and have identified parameters and novel biomarkers for monitoring MOD. In the future, with further study, biomarkers including serum and urinary NGAL, cystatin C and IL-6 show good ability to predict AKI and may be useful in long-term follow up. Cardiology assessment including measurement of serum troponin, pro-NT-BNP, echocardiography using tissue doppler and speckle-tracking may be used to monitor cardiovascular function in children with CP. The use of these biomarkers may initially prove most useful to the research community in providing new insights in to subtle organ dysfunction. However, this may lead to discovery of new therapeutic targets and improved outcomes. There is also the possibility that these biomarkers will become the norm in clinical care if there are clinically-relevant long-term consequences noted in those areas.

Throughout this article, where possible, we have attempted to comment on the impact of dysfunction in each of the organ systems involved. For example, pain is one of the most significant consequences of dysfunction in the gastrointestinal system. It is also an example of the complex interactions between the neurological and non-neurological systems which may receive further attention by considering CP as a multi-system disorder with multi-organ dysfunction. However, in many cases, the answer to that question has not been adequately addressed in the literature. We hope that this article highlights some of those gaps and provokes further research into outcomes in the less well-studied organ systems of children with CP.

An example of an area where further research and exploration of consequences is required is the immune system. Children with CP have altered immune function compared to age-matched controls. Understanding the immune response in these children with CP and exploring systemic inflammation holds promise for future development of immunomodulatory adjunctive therapies. Further research in this area can contribute to better prediction of outcome and improved prognosis.

We believe that a MOD scoring system would prove useful, perhaps especially in the research context as a way to correlate with other health related outcomes. It may also prove useful in allowing advanced clinical planning, discussions around prognosis and follow-up of children with CP throughout the lifespan. CP registries are now in existence throughout the world. The information provided by these registries, as well as the expert collaboration that they promote, in conjunction with a MOD scoring system, might allow for the production of tailored guidelines for follow-up and management of MOD in children with CP. We suggest that follow up of multi-organ function is important to identify and pre-emptively manage potential long-term complications in CP.

Another issue which may be raised, if one considers CP as a multi-system disorder, is the definition of CP. The definition developed by Rosenbaum et al. (1) which was published in 2007 is probably the most widely accepted. However, recently questions have arisen regarding whether this definition needs to be revisited in light of increasing knowledge in several areas, for example, the genetic causes and predispositions to development of CP (270, 272–274). The Rosenbaum definition is limited in its reference to co-morbidities stating that “The motor disorders of cerebral palsy are often accompanied by disturbances of sensation, perception, cognition, communication, and behavior, by epilepsy, and by secondary musculoskeletal problems” (1). If the definition of CP is revisited, consideration may be given to including a reference to the multi-system nature of the disorder.

This review highlights the need for an awareness amongst healthcare professionals of the multi-systemic complications that are associated with CP, particularly in those with greater levels of impairment. That many of these children communicate non-verbally compounds the difficulties that the physician faces in understanding the source of many of their symptoms. The cause of symptoms such as pain, behavioral or sleep disturbance may be multi-factorial and present substantial diagnostic challenges. A step-wise, logical, multi-system approach, with support from a multi-disciplinary team, is required to ensure that the best care is provided to these children and their families.

## Author Contributions

JA contributed to the conception and design, acquisition, analysis and interpretation of data, drafted the first and subsequent drafts of the work, revised it critically, and approved the final version of the manuscript. ZZ, SD, LW, and ZA contributed to the conception and design, acquisition, analysis and interpretation of data, drafting the work and revising it critically, and approved the final version of the manuscript. MS, OF, AG, AJ, TL, SQ, BE, JR, SP, PK, ER, CM, LB, OH, LG, and SK contributed to the analysis and interpretation of data, revising the work critically, and approved the final version of the manuscript. DM and EM contributed to the conception and design, analysis and interpretation of data, drafting the work and revising it critically, and approved the final version of the manuscript. All authors contributed to the article and approved the submitted version.

## Conflict of Interest

The authors declare that the research was conducted in the absence of any commercial or financial relationships that could be construed as a potential conflict of interest.

## Publisher's Note

All claims expressed in this article are solely those of the authors and do not necessarily represent those of their affiliated organizations, or those of the publisher, the editors and the reviewers. Any product that may be evaluated in this article, or claim that may be made by its manufacturer, is not guaranteed or endorsed by the publisher.
